# Apoplexy

**Published:** 1888-12

**Authors:** C. O. Weller


### APOPLEXY.

By C. O. Weller, M. D.

[Read at Travis County Medical Society Meeting, and Ordered Printed.]

THE SUBJECT that I have to present for your consideration to-night, is uræmia and apoplexy. As neither word conveys the idea of a specific disease in itself, but is only used to designate a certain condition of the body, it will become necessary to enter briefly into an investigation of the causes productive of each. By uræmia we understand that an unhealthy condition of the blood exists, resulting from retention in it, of certain constituents excrementitious in their nature, that should be eliminated by the kidneys. These excrementitious matters, are those, resulting from the decomposition of nitrogenous matters in the body, and are urea, creatinine, sodium and potasium urates, and sodium and potassium hippurates. Of these, urea is by far the most important constituent of the urine, both in regard to character and amount, forming more than one-half the entire quantity of its solid ingredients, and (80) eighty per cent. of all those of an organic nature. The most important fact known with regard to the origin of urea is, that it is *not* found in the kidneys, but pre-exists in the blood in small proportion, and is drained away from the circulating fluid during its passage through the renal vessels. This was first shown by the experiments of Prevost and Dumas, who after extirpating the kidneys or tying the renal arteries in the living animal, found the quantity of urea in the blood increased in marked proportion, owing to the arrest of its elimination by the kidneys. It has also been found in the blood of the human subject in cases of renal diseases in nearly ten times its normal quantity. It has *not* been found, however, in sufficient quantitiy in any of the solid tissues to indicate the immediate source of its production. It is either formed in the blood itself, by transformation of some previous nitrogenous combination, or it is absorbed by the blood too rapidly to be detected as an ingredient of the solid tissues. The retention, then, of these substances of nitrogenous origin in the blood, brings about that morbid condition that we recognize as uræmia.

The diseases productive of this state, are those grouped together under the general name of Bright's disease. It is also a result of the pregnant state. There are three forms of this, constituting really three distinct and separate diseases, having some symptoms in common, but differing essentially in the pathological condition of the kidney. These are nephritis, granular, degeneration, and lardaceous disease. It is not however with in the scope of this paper to enter into a detail of the structural changes of these different forms of Bright's disease, or differentiate their pathology. It is sufficient for our present purpose, to know that they are all factors in producing that morbid condition known as uræmia, by interfering with the normal functions of the kidneys. And not only is the condition that we designate uræmia, wherein the nervous system appears to be|the chief sufferer, a sequent to these diseases, but there is, as a result of them a general depravation of the blood, and a tendency to numerous conflicting troubles; such as pericarditis, pleuritis, peritonitis, pneumonitis, bronchitis, neuralgia, amaurosis, hypertrophy of the left ventricle, and cerebral hemorrhage. Hypertrophy of the heart, especially of the left ventricle, is one of the most common results of this blood dyscrasia. Dr. Bright thought this was due to the altered condition of the blood, rendering it difficult of passage through the capillaries, consequently the *vis a tergo* has to be augmented, by more powerful contractions of the left ventricle, and which finally eventuates in its hypertrophy. After a patient has suffered a graeter or less length of time from organic disease of the kidneys, and he is not carried off by some of the complications that may intervene, the nervous system begins to manifest the influence of the morbid condition of the blood on it. The patient begins to experience a sense of restlessness, giddiness, or stupidity; he may have wandering and delirium, and after a time, convulsions may ensue, or he may pass into a quiet stupor. This state of quiet stupor is characteristic of renal disease. The pulse generally is quiet, the skin cool, the temperature about normal, the pupil sometimes normal, and sometimes dilated. There is a peculiar stupor, which increases as the end draws near, and the insensibility deepens. The respiration is generally quick, and accompanied with labial rather than gutteral sounds.

There is no localized paralysis, no inequality between the two sides, but general immobility and torpor. "The lateral symmetry of uræmia is one of its most important diagnostics. The condition of the brain under such circumstances is one which may be briefly described." The noticeable fact about it, is extreme anæmia. The large vessels are empty, the gray matter blanched, while the white matter is perfectly colorless, scarcely a trace of blood exuding upon the cut surface. There is often slight excess of watery fluid in the various cavities, and interstices, but not sufficient to produce any pressure upon the cerebral substance. "The brain is generally firm as in health."

The prognosis in uraemia is generally unfavorable. It depends principally on the extent of the kidney disease, of which it is a result. When co-existing with acute renal trouble, the prognosis is more favorable than when with the chronic. "Puerperal cases, although involving great immediate danger, are very frequently recovered from, probably because they owe their origin to a combination of circumstances, which do not long persist. The leading indication for treatment is to restore the suppressed secretion of the kidney. For this purpose non-irritating diuretics, as digitalis, and copious drafts of pure water should be used. If we should fail in this, we must assist the kidney by the administration of hydragogue cathartic, and diaphoretics When convulsions occur, chloroform, the bromides and hydrate chloral, must be used, singly or combined according to the indications in each case.

Apoplexy, like the foregoing, is not in itself a definite disease. It only represents a condition that may result from different pathological causes. As commonly used, it is applied to the sudden abolition, or not at all impairment of consciousness and power of motion. "The apoplectic condition may be due to a sudden cerebral lesion, such as hemorrhage or vascular obstruction; or to a sudden shock, or other impression arresting the cerebral functions, but causing no visible alteration in the brain. The great cause of apoplexy is sudden cerebral lesion, which may be traumatic, or without external injury. Injury may lead to apolexy by simple concussion, by laceration of the brain, or by rupture of vessels and hemorrhage. Apoplexy not due to injury may be caused by congestion; by thrombosis or embolism; but especially by hemorrhage. The latter is its most common and most efficient cause. Profound coma is rarely due to any other spontaneous cerebral lesion." In fatal cases by far the most frequent of these conditions is hemorrhage within the cranium. "The abruptness of an attack of apoplexy is implied in the name. In the majority of cases, it is without premonition; though it is sometimes preceded by certain cerebral symptoms, as weight or fulness, vertigo, tinnitus aurium, and flushing of the face. These symptoms are not, however, sufficiently significant to warrant the prediction of an impending attack, except they be in a patient who has been the subject of a previous one.

It is not rare for the stroke to occur at a moment when the patient feels unusually well. One of the prominent symptoms of apoplexy is loss of consciousness, without obvious failure of the heart's action. The onset is often instantaneous so that the sufferer falls to the ground The face may be flushed or pale,—it is rarely very pale. The heart and arteries beat, often, with undue force and frequency. Respiration continues, but is labored and stertorous. The limbs are motionless. In severe cases no reflex action can at first be excited. The pupils may be dilated, contracted or unchanged; in profound coma, they are usually dilated, and they often vary in size spontaneously, being sluggish in their action to light. The full development of the apoplectic state may occupy a few seconds or be protracted to half an hour or more. The liability to apoplexy has a manifest relation to age; which increases from twenty years upward, and in the majority of cases the age is over fifty. Cases under twenty are rare, yet they are occasionally observed. Microscopical researches have appeared to show that hemorrhages into the substance of the brain is generally a result of fatty or calcareous degeneration of the coats of the smaller cerebral arteries. Sometimes, also small aneurismal dilatations of minute arteries are found. Softening of the cerebral substance sometimes precedes the hemorrhage. This softening is due to degeneration of the arterial coats, or perhaps to the interruption of the circulation by an embolus. The dependence of extravasation on this condition of the minute arteries of the brain explains the fact that apoplexy is most likely to occur in advanced life, the degenerative changes just named occuring especially in old age.

The prognosis in apoplexy depends upon the extent of the brain lesion and intensity of the attack. As long as unconsciousness is complete and reflex action abolished, the patient is in danger of speedy death. Persistent depression of temperature, or a rise of several degrees above the normal, after an initial fall, are both of grave significance. A sudden recurrence or increase of apoplectic symptoms, a few hours or days after a slighter attack, is always grave, indicating a fresh extravasation. If the localizing symptoms point to a lesion of the pons or medulla, the prognosis is very unfavorable. Previous cerebral disease renders the prognosis bad. This is also unfavorable, when the organic functions of the circulation, respiration and deglutition, are notably impaired. Early return of consciousness and alteration in temperature are favorable signs. An early recovery from the hemiplegic state is also a favorable symptom. A complete recovery from the hemiplegia, however, is rare. Apoplexy with this condition is followed by more or less impairment of mind. A remarkable sequel in some cases is the loss of speech, not from difficulty of articulation, but from an inability to use words. The cases of complete recovery from the apoplectic state, are probably those due to congestion rather than extravasations.

I will only say a few words about treatment. If absence of hemiplegia, and the presence of a flushed face and strong resisting pulse indicate congestion as the cause, prompt and free venesection is indicated, and upon it may depend the life of the patient. An emetic and purgative are also indicated, if there is evidence of the stomach and bowels being loaded. If the evidences of extravasation are present, the propriety of blood letting is doubtful. It will not remove the clot. It is contra-indicated by feebleness of constitution, completeness of coma indicating a large extravasation, or if its situation at the base of the brain be indicated by notable disturbance of respiration and at least deglutition. Good judgment is required in the use of this remedy. It will be pernicious or useful according to the discrimination with which it is employed. In view, however, of the fact that apoplexy generally involves extravasation, this measure is indicated in only a small proportion of cases. The patient should be placed in a cool, airy apartment, the head elevated, and cool applications made to it, whilst the feet are immersed in hot mustard water, if they are cool. If the patient survive the immediate effects of the first attack, remedies must be addressed to the conditions that may remain, as the hemiplegic state, local inflamation that be may excited by the clot, etc.

As to the differential diagnosis of uræmia and apoplexy in a given case, it may be made without great difficulty, provided the uræmia is not associated with cerebral hemorrhage, and we can get a correct history of the case. If called to a patient in a comatose state, we must take into consideration his antecedents, and if we find he has been a sufferer from renal desease, or the coma has come on gradually, or has succeeded convulsions, the complexion anænic, the pulse quiet, the temperature normal or below, the stertor labial instead of gutteral, if there is no localized paralysis, we may feel quite sure we have a case of uræmic coma. If, however, there should be hemiplegia, we may very reasonably conclude we have apoplexy resulting from cerebral hemorrhage. But these conditions may exist. One of the common terminations of the granular degeneration of the kidneys, with uræmia, is in cerebral hemorrhage, and where these are associated we must rely on the history of the case and analysis of the urine to establish our diagnosis. If we cannot get a satisfactory history of the patient, and all other causes that might produce the comatose state have been excluded, and we are to decide between uræmic intoxication and apoplexy, if paralysis be present and the composition of the urine is normal, the evidence is in favor of apoplexy. If there should be albuminous urine, with renal casts, and no paralysis, it would favor uræmia, but if both albuminous urine and paralysis are present, it would be probable that uræmia and apoplexy from cerebral hemorrhage exists.

Apoplectic attacks are generally sudden, frequently coming on when the patient is feeling unusually well; uræmic coma is gradual, and often associated with accumulations in the serous cavities as well as general anasarca. In apoplexy the patient is often of robust form and florid complexion, though by no means always, whilst in uræmia he is anæmic and pale. The character of the coma will sometimes help us to decide. In uræmia, it is less profound than in cerebral mischief. In apoplexy the coma is often very profound. The mode of onset has some significance. In apoplexy it is sudden, in uræmia slow. The uræmic patient becomes drowsy, and then comatose. But with convulsions, uræmic coma may also come on suddenly. General convulsions at the outset favor uræmia. Cerebral mischief sometimes commences with convulsions, but the convulsions then are generally unilateral, and one-sided symptoms are almost always afterwards to be recognized. Rigidity of limbs, or local muscular twitching, is, if constant in seat, in favor of cerebral mischief; if variable in position, it is in favor of uræmia. The state of the pupils is alone of little importance. The pupil may be normal or dilated in uræmia or apoplexy. Inequality of the pupils, an unilateral symptom, points to brain mischief. In a doubtful case, the retina should be examined, since albuminuric retinitis points, in the absence of the signs of localized cerebral lesion, strongly to uræmia. Lastly, the temperature should be noted. In uræmia there is persistent uniform depression; in cerebral lesions the initial depression is succeeded by a rise to a point above the normal.

Before closing I must say I am aware of the incompleteness of the treatment of this subject, but for whatever of merit the paper may contain, I am indebted to Messrs. Dickinson, Grainger, Stewart, and Gowers, of Great Britain, and Dalton, Flint, and Bartholow, of New York and Philadelphia.

